# Questioning Cause and Effect: Children with Severe Asthma Exhibit High Levels of Inflammatory Biomarkers Including Beta-Hexosaminidase, but Low Levels of Vitamin A and Immunoglobulins

**DOI:** 10.3390/biomedicines8100393

**Published:** 2020-10-06

**Authors:** Amali E. Samarasinghe, Rhiannon R. Penkert, Julia L. Hurwitz, Robert E. Sealy, Kim S. LeMessurier, Catherine Hammond, Patricia J. Dubin, D. Betty Lew

**Affiliations:** 1 Department of Pediatrics, University of Tennessee Health Science Center, Memphis, TN 38163, USA; amali.samarasinghe@uthsc.edu (A.E.S.); klemessu@uthsc.edu (K.S.L.); chammo17@uthsc.edu (C.H.); pdubin@uthsc.edu (P.J.D.); dlew@uthsc.edu (D.B.L.); 2 Department of Microbiology, Immunology and Biochemistry, University of Tennessee Health Science Center, Memphis, TN 38163, USA; 3 Children’s Foundation Research Institute, Le Bonheur Children’s Hospital, Memphis, TN 38103, USA; 4 Department of Infectious Diseases, St. Jude Children’s Research Hospital, Memphis, TN 38105, USA; rhiannon.penkert@stjude.org (R.R.P.); bob.sealy@stjude.org (R.E.S.); 5 Institute of Molecular Biology, University of Oregon, 1318 Franklin Blvd, Eugene, OR 97403, USA

**Keywords:** beta-hexosaminidase, retinol binding protein, immunoglobulin, periostin, surfactant protein-D, receptor for advanced glycation end products

## Abstract

Asthma affects over 8% of the pediatric population in the United States, and Memphis, Tennessee has been labeled an asthma capital. Plasma samples were analyzed for biomarker profiles from 95 children with severe asthma and 47 age-matched, hospitalized nonasthmatic controls at Le Bonheur Children’s Hospital in Memphis, where over 4000 asthmatics are cared for annually. Asthmatics exhibited significantly higher levels of periostin, surfactant protein D, receptor for advanced glycation end products and β-hexosaminidase compared to controls. Children with severe asthma had lower levels of IgG1, IgG2 and IgA, and higher levels of IgE compared to controls, and approximately half of asthmatics exhibited IgG1 levels that were below age-specific norms. Vitamin A levels, measured by the surrogate retinol-binding protein, were insufficient or deficient in most asthmatic children, and correlated positively with IgG1. Which came first, asthma status or low levels of vitamin A and immunoglobulins? It is likely that inflammatory disease and immunosuppressive drugs contributed to a reduction in vitamin A and immunoglobulin levels. However, a nonmutually exclusive hypothesis is that low dietary vitamin A caused reductions in immune function and rendered children vulnerable to respiratory disease and consequent asthma pathogenesis. Continued attention to nutrition in combination with the biomarker profile is recommended to prevent and treat asthma in vulnerable children.

## 1. Introduction

Asthma affects approximately 340 million people worldwide and is the leading noncommunicable lung disease in children [1]. Because asthma is a multifactorial syndrome, there is a heterogenous response to current controller therapies such as corticosteroids. Biologics such as anti-immunoglobulin (Ig)E (omalizumab) [2] and anti-interleukin (IL)-5 (mepolizumab) [3] are used to treat severe asthmatics, although their efficacy is variable [4,5]. More recently, additional biologics such as IL-5 receptor-alpha blocker (benralizumab) and IL-4 receptor-alpha blocker (dupilumab) have been approved for treatment of patients with moderate to severe eosinophilic asthma [6]. In addition to heterogeneous endotypes, variations in triggers (both environmental and psychological) and socioeconomic factors, including poor access to care, further complicate asthma management. The discovery of new biomarker profiles that correlate with disease severity in children may help customize treatments and provide better long-term care to patients. 

Of the 26.1 million asthmatics in the United States [7], incurring an economic burden of approximately $81.9 billion annually [8], approximately 23.4% are children [7]. While these patients are dispersed across the country, some regions within each state have higher incidence [7]. The Asthma and Allergy Foundation of America ranked Memphis, Tennessee (TN) as the second worst city to live in with allergies in the 2016 national ranking [9]. The TN Department of Health identified Shelby County (which includes Memphis) as the county with the greatest childhood asthma burden in the state [10]. The economic hardship indexes in some Shelby county cities including Memphis are among the highest in the country. Wealth disparities are also apparent with the median household income being 42% lower for African Americans compared to whites [11]. Low socioeconomic status is associated with poor nutrition and chronic diseases including asthma [12]. African Americans account for more than 85% of adult asthmatics in TN [13,14]. The asthma clinic at Le Bonheur Children’s Hospital in Memphis cares for over 4000 children annually. The majority of these children are African American [15] and approximately 4.5% of these children require intensive care at some point during their disease course. Therefore, as a city high in wealth and health disparities, identification of biomarkers that may be informative of this patient population is important to clinical decision making.

Poor nutrition has been correlated with poor immune responses to pathogens [12]. In Memphis, low vitamin A levels correlated with low antibody responses toward an influenza virus vaccine in children [16]. Moreover, vitamin A insufficiencies/deficiencies were associated with poor outcomes among children hospitalized with respiratory viral infections [17] suggesting that correlations may occur between nutrients and other immune conditions like asthma. Our access to a large cohort of predominantly African American pediatric patients with severe asthma provided an opportunity to expand the peripheral blood marker profile. We questioned whether pediatric patients with severe asthma had abnormal vitamin A, immunoglobulin, cytokine/chemokine and β-hexosaminidase (HEX) levels, and if these factors were interrelated. Altogether, our evaluations were performed to address cause and effect relationships while defining targets for better prophylaxes, diagnostics, and therapeutics to help reduce the severe consequences of asthma in children.

## 2. Materials and Methods

### 2.1. Study Participants and Sample Collection

One hundred patients with severe asthma from the asthma clinic in Le Bonheur Children’s Hospital were enrolled. All asthma patients enrolled in this study were followed by board certified allergists and pulmonologists. Hospitalized, nonasthmatics (*n* = 47) served as controls. The study was approved by the Institutional Review Board of the University of Tennessee Health Science Center (11-01245-XP). Diagnoses of severe asthma were based on the World Health Organization (WHO) consensus on severe asthma [18]. Inclusion criteria for severe asthmatic patients consisted of an asthma-related intensive care unit (ICU) admission, a minimum daily requirement of inhaled corticosteroids of 800 µg for greater than six months, chronic systemic steroids (>0.5 milligram per kilogram [mg/kg], every other day) for six months, or at least three short courses of oral steroids per year (1–2 mg/kg/day in a twice daily divided dosing schedule, maximum dose 30 mg twice a day [bis in die, BID], for five days/each course). Exclusion criteria were the presence of another chronic lung disease or the use of chronic steroid therapy for another disease [15]. Five samples from the asthma group were excluded from analyses due to insufficient plasma volume or due to the patient’s postpartum status. Controls were hospitalized due to a variety of diseases/conditions, excluding asthma. Plasma isolated from collected blood samples were aliquoted and frozen at −80 °C until use. 

### 2.2. Determination of β-HEX and Other Immune Mediators in the Plasma 

β-HEX activity was measured by colorimetry as previously described [19]. Briefly, a sample of 25 µL plasma was incubated with 50 µL prewarmed substrate (4-nitrophenyl-*N*-acety-beta-d-glucosaminide, 5 mM, pH 4.5) for 60 min at 37 °C in duplicate and the enzymatic reaction was stopped by 50 µL of 0.1 M NaOH. The p-nitrophenyl released by the enzymatic hydrolysis of the substrate was quantified using an enzyme-linked immunosorbent assay (ELISA) reader at 405 nm wavelength. Reagents were purchased from Sigma-Aldrich (St. Louis, MO, USA). Absorbance was converted into micromoles of substrate cleaved using the Beer-Lambert Law [Molar concentration = Abs405 nm/E × l (light path in cm)] molar extinction coefficient for p-nitrophenol (E = 18,700). International units of specific activity were defined as micromoles of substrate cleaved per hour per liter of plasma. Other predetermined inflammatory mediators in lung disease were measured with an 18-plex multiplex assay (R&D Systems, Minneapolis, MN, USA) with a Luminex MAGPIX^®^ Instrument with xPONENT software (Luminex, Austin, TX, USA). In rare instances, a cytokine/chemokine measurement could not be obtained and was omitted. We performed a two-fold dilution of the samples prior to the multiplex assay, after which scores were dilution-corrected. When values were above or below the assay’s LOD, they were assigned the LOD value for comparison purposes.

### 2.3. Determination of Immunoglobulin Levels in Plasma

Immunoglobulin (Ig) M, IgG subclasses 1–4, IgA and IgE were quantified from plasma using a bead-based multiplex immunoassay (Millipore Sigma, Billerica, MA, USA) with a Luminex 200 Multiplex Instrument and xPONENT software. Ig concentrations were determined using Milliplex Analyst software (Millipore Sigma, Billerica, MA, USA). The LOD was substituted for subclass values that fell above/below the LOD thresholds. 

### 2.4. RBP Assay

Plasma RBP was used as a surrogate measure for vitamin A (retinol) [20]. Levels of RBP were measured by ELISA using an R&D Systems human RBP4 Quantikine kit (R&D Systems, Minneapolis, MN, USA). The precise cut-offs for vitamin deficiencies and insufficiencies remain a topic of continued debate [20,21,22,23]. Herein, we defined vitamin A deficiency as RBP <15,000 ng/mL (approximately <0.7 μmol/L) and insufficiency as ≥15,000 ng/mL but <22,000 ng/mL RBP (approximately ≥0.7 μmol/L but <1.05 μmol/L). 

### 2.5. Statistical Analyses

Medians in each group were calculated. Statistical tests included Mann-Whitney U and Spearman rank-order correlation tests. In several instances (e.g., for cytokines/chemokines), there were numerous values that scored above or below the LOD, in which case the Fisher’s exact test was used to compare frequencies of high/low values within patient populations. Calculations were performed using GraphPad Prism software (Versions 7-8, Graphpad, San Diego, CA, USA).

## 3. Results

### 3.1. Patient Demographics

One hundred patients with severe asthma (three to 18 years of age) from the asthma clinic in Le Bonheur Children’s Hospital were enrolled along with 47 hospitalized, nonasthmatics in the same age range. 

This pilot, observational, cross-sectional study was approved by the Institutional Review Board of the University of Tennessee Health Science Center and patient enrollment occurred between June 2011 and December 2014. Out of 104 eligible asthmatics, 100 patients/parents agreed to participate in the study. Among asthmatics, 97% were atopic [15]. Five samples from the asthma group were excluded from analyses due to insufficient plasma volume or due to the patient’s postpartum status.

Participant characteristics are shown in Table 1. The nonasthmatic patients were hospitalized due to a wide variety of diseases/conditions. Diagnoses included inflammatory or noninflammatory infections (local or systemic) or noninfectious conditions including diabetic ketoacidosis, acute gastroenteritis and dehydration, cellulitis, fever, sepsis, meningitis, pneumonia, chest pain, indwelling venous catheter malfunction, ataxia, myositis, hip pain, osteomyelitis, cerebrovascular accident, seizure, drug ingestion, Henoch-Schönlein purpura, and constipation. 

Nonasthmatics included approximately equal numbers of males and females, whereas asthmatics were predominantly male. Patients in both groups were primarily African American, with a bias toward African Americans among asthmatics (as was previously observed among adults [14]). A number of medications/treatments were common in asthmatics at the time of enrollment. Most asthmatics were on oral/inhaled corticosteroids, while some asthmatics were also receiving allergen immunotherapies or omalizumab. Formulations of inhaled corticosteroids were budesonide (800 micrograms [mcg] for all ages) and fluticasone (>440 mcg for ages six to twelve years and 880 mcg for ages >12 years). Smoke exposure, including active and passive exposure, was reported by 22 of 95 asthmatics. There was no attempt to exclude active smokers from the study. 

### 3.2. Cytokines/Chemokines Mark Severe Asthma in Children 

**Figure 1:**
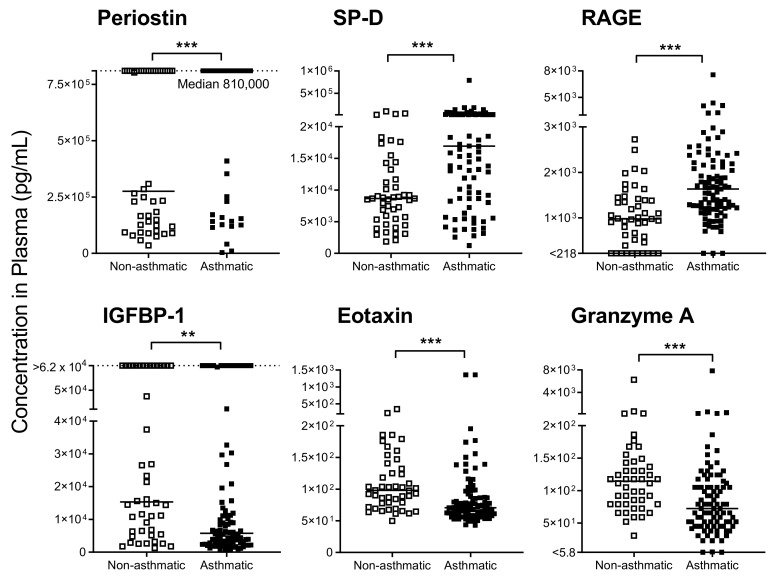
Immune mediators in plasma were altered in asthmatics compared to hospitalized nonasthmatics. Significant differences were observed in plasma immunomodulators between asthmatics and controls. Dotted lines are indicative of upper limits of detection (LOD). Solid lines show the median in each group. For surfactant protein-D (SP-D), receptor for advanced glycation end products (RAGE), eotaxin and granzyme A, data were analyzed by Mann-Whitney U tests with significance marked by *** *p* < 0.001. For periostin, the Fisher’s exact test was used to compare patient populations for scores above/below the upper LOD (*** *p* < 0.001). For insulin growth factor binding protein (IGFBP-1), the Fisher’s exact test was used to compare patients for scores above/below 5000 pg/mL (** *p* < 0.01).

When blood cytokines/chemokines were quantified (Figure 1), several factors showed striking differences between asthmatic and hospitalized, nonasthmatic children. Examples included periostin, surfactant protein-D (SP-D), and receptor for advanced glycation end products (RAGE). These factors were found to be significantly elevated in the severe asthmatics compared to controls (*p* < 0.01 or *p* < 0.001, Mann Whitney or Fisher’s exact tests, see Figure 1 legend). In contrast, insulin growth factor binding protein (IGFBP)-1, eotaxin, and granzyme A were significantly reduced in asthmatic compared to nonasthmatic patients (*p* < 0.01 or *p* < 0.001, Mann Whitney or Fisher’s exact tests, see Figure 1 legend). Not shown are results from additional tested factors including erythropoietin, interferon (IFN)β, IFNγ, IL-5, IL-13, IL-17α, IL-22, IL-33, IGFBP-3, transforming growth factor α, granzyme B and amphiregulin, that showed slight or no differences between groups. 

Differences between test and hospitalized control groups may have been due to elevated biomarker levels in the hospitalized controls (sick nonasthmatic children). We, therefore, identified values in asthmatic patients that differed both from (i) hospitalized controls and (ii) reference ranges established for healthy children [24,25,26,27]. Of particular interest were periostin and IGFBP-1, both of which were abnormal in asthmatics. Specifically, periostin was extremely high in asthmatics; the median score for this population was at the assay’s upper limit of detection (LOD, 810,000 pg/mL) and was well above the healthy pediatric reference range described by Caswell-Smith et al. [27]. In contrast, most asthmatics exhibited IGFBP-1 values that were below the healthy pediatric reference range (Quest Diagnostics, lower limit 5000 pg/mL).

### 3.3. High β-HEX Levels in Severe Asthmatics

**Figure 2:**
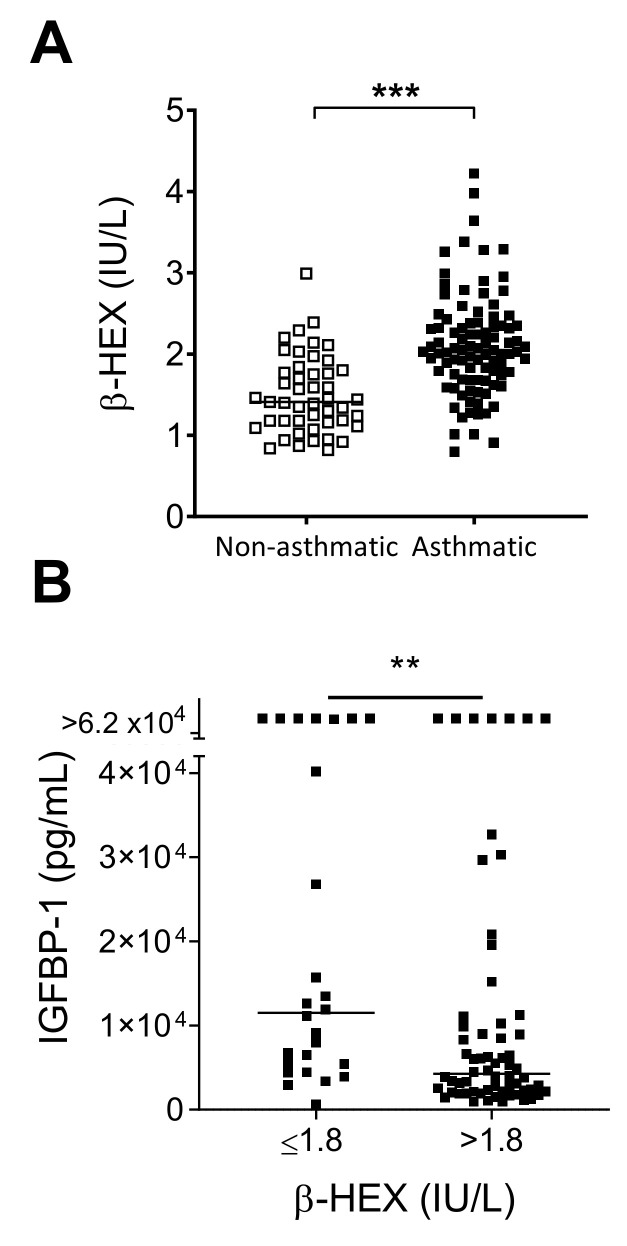
β-hexosaminidase (β-HEX) in asthmatic patients. (**A**) Asthmatic children had higher levels of β-HEX compared to hospitalized, nonasthmatics. Medians in each group are shown by horizontal lines. Data were analyzed by the Mann-Whitney U test (*** p < 0.001). (**B**) High β-HEX levels associated with low IGFBP-1. Asthmatic patients with >1.8 IU/L β-HEX exhibited lower levels of IGFBP-1 compared to asthmatic patients with ≤1.8 IU/L β-HEX values. Patients in the two groups were compared for high/low IGFBP-1 values (cut-off 5000 pg/mL) using the Fisher’s Exact test (** p < 0.01).

Our cohort of severe asthmatics had significantly increased levels of β-HEX when compared to nonasthmatic, hospitalized patients (Figure 2A). The plasma/serum range in healthy children for this protease-resistant enzyme has not been reported to date, but levels tend to increase with age with a mean of approximately 2.5 IU/L in healthy adults [19,28]. 

We asked if β-HEX values were associated with any of the factors shown in Figure 1. We assigned asthmatic patients to two groups based on β-HEX levels ≤ or >1.8 IU/L and then found that higher β-HEX was associated with lower IGFBP-1 (Figure 2B). Patients with >1.8 IU/L β-HEX were also more likely to have detectable IL-5 and IL-13 (Fisher’s exact test, *p* < 0.005), two cytokines that were jointly expressed by patients in our study. Of the 95 asthmatic patients, fourteen had both IL-5 and IL-13 levels above background, and all of these patients were in the group with >1.8 IU/L β-HEX.

### 3.4. Children with Severe Asthma Have Low Plasma IgG and IgA Levels

**Figure 3:**
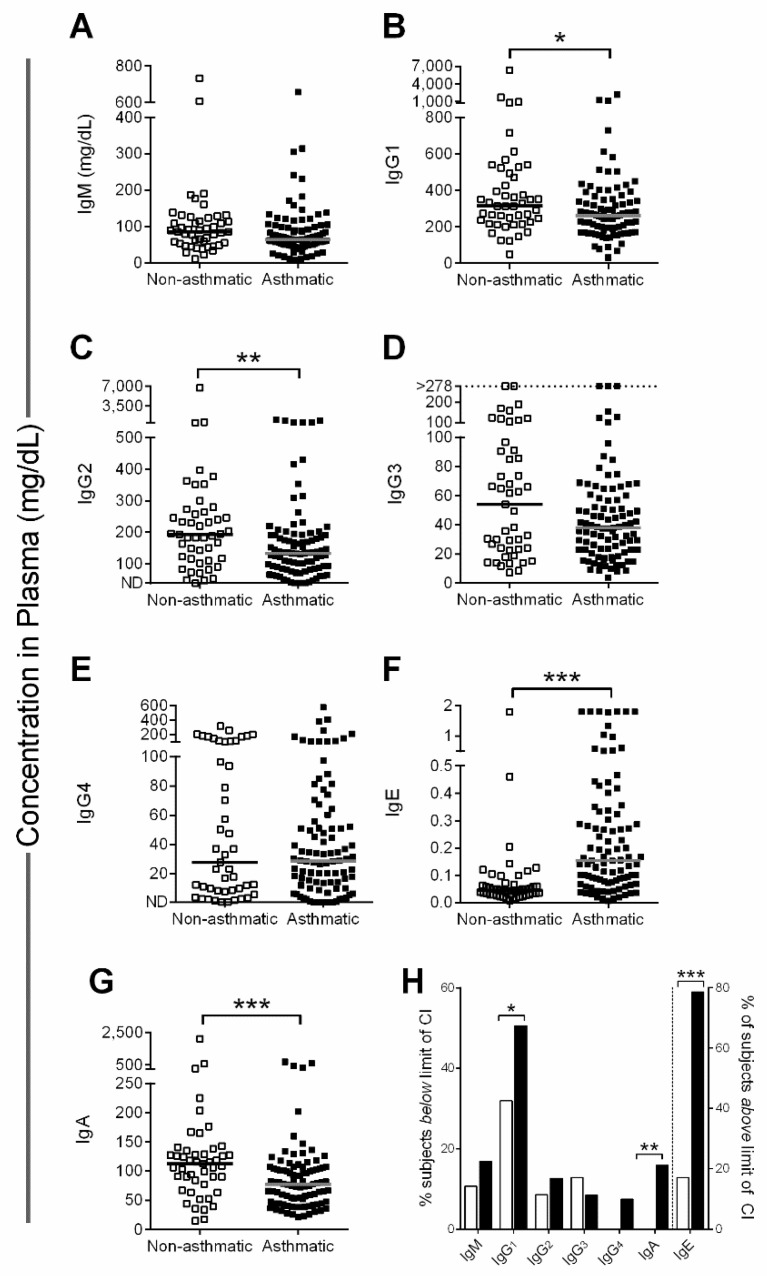
Low immunoglobulin (Ig) levels in severe asthmatics. (**A**–**G**) Lines show the median in each group. One value for IgE (not shown) in the asthmatic patient group was >3 mg/dL (>12,500 kU/L). (**H**) % subjects outside age-specific immunoglobulin reference range (95% confidence interval [CI]), either below 95% CI (bars to the left of the dotted line) or above 95% CI (bars to the right of the dotted line). Clear and black bars represent control and test patients, respectively. Data were analyzed by the Mann-Whitney U test with significance indicated by * *p* < 0.05, ** *p* < 0.01, and *** *p* < 0.001.

We measured IgM, IgG, IgE and IgA isotypes in our asthmatic patient and control groups (Figure 3). IgM, IgG3, and IgG4 (Figure 3A,D,E) were equivalent between the two groups, whereas IgG1 (Figure 3B), IgG2 (Figure 3C), and IgA (Figure 3G) levels were significantly lower in asthmatics compared to nonasthmatic patients. 

IgE levels (Figure 3F) were significantly higher in the asthmatics. A small number of patients (*n* = 6) received omalizumab and showed a broad range of IgE values (0.04–3.65 mg/dL, median value of 1.038 mg/dL; the effect of omalizumab on assay results was not determined). 

IgG1, the most abundant blood isotype, fell below age-specific reference ranges among 51% of severe asthmatics (Figure 3H, see Supplementary Materials Table S1 for immunoglobulin age-specific reference ranges from the Mayo Clinic). IgA and IgG2 were lower than age-specific reference ranges in 16% and 13% of severe asthmatics, respectively. IgE, a classical marker of allergy, was elevated above the age-specific reference range in the majority of severe asthmatics (Figure 3H). IgG1 levels often increase with age in healthy children, but we did not observe these increases among asthmatics (Supplementary Materials Figure S1).

### 3.5. Low Plasma RBP Levels Correlate with Low IgG1 in Severe Asthmatics

**Figure 4:**
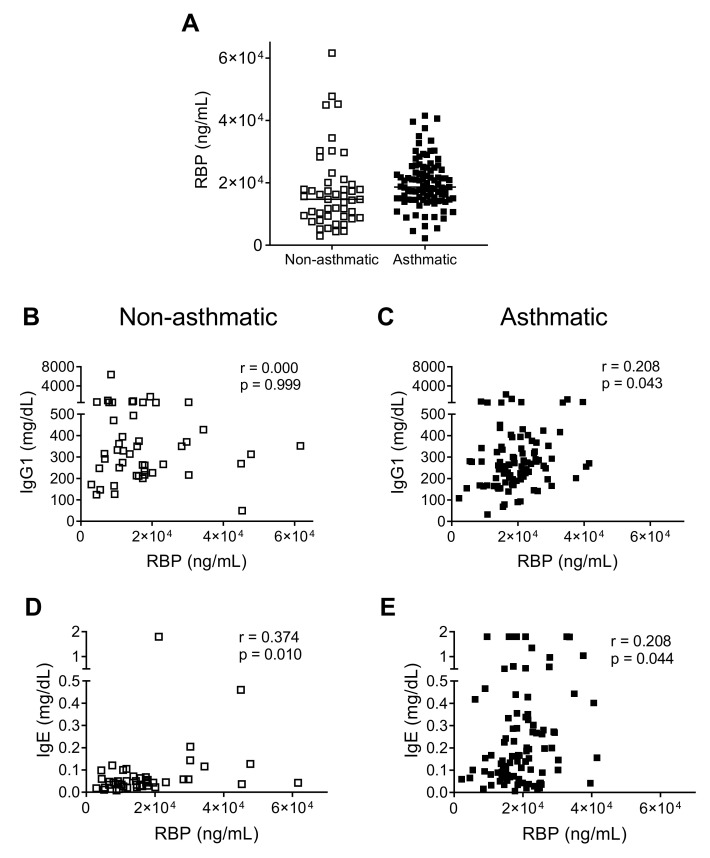
Correlation between retinol binding protein (RBP) and immunoglobulin (Ig). (**A**) RBP levels were compared between the two patient groups. Lines show the median in each group. (**B**,**C**) Asthmatics (but not controls) had moderate positive correlations between RBP and IgG1. Data were analyzed using the Spearman rank-order correlation test. (**D**,**E**) Spearman rank-order correlation analyses showed that both controls and asthmatics exhibited positive and moderate correlations between RBP and IgE.

The majority of children in both patient groups were deficient/insufficient in vitamin A as defined by a retinol binding protein (RBP) level of <22,000 ng/mL (Figure 4A) [20]. For each of the patient groups, we independently compared RBP measurements to published RBP data from 79 healthy children in the Memphis area [16]. The RBP values between severe asthmatics and hospitalized children were not significantly different, but RBP values in each group were significantly lower than those of the previously-described healthy children [16] (Mann-Whitney U test, *p* < 0.001). This was despite the fact that the latter group included a subset of children who exhibited vitamin A deficiencies/insufficiencies. 

Since low levels of vitamin A have been associated with poor immune responses [16,29,30,31,32,33,34], we examined correlations between RBP and total plasma immunoglobulin levels. We observed a moderate positive correlation between RBP and IgG1, the most abundant antibody in plasma, in asthmatic, but not in control patients (Figure 4B,C). Similar to IgG levels, RBP levels did not increase significantly with increasing age among the group of asthmatic children (Supplementary Materials Figure S2). Among both patient groups, there was a positive correlation between RBP and IgE, although IgE levels were much higher in asthmatics (Figure 4D,E).

Finally, we asked if β-HEX correlated significantly with RBP and/or total plasma immunoglobulin levels. We found a negative correlation between β-HEX and serum IgG2 in asthmatic patients (Spearman rank-order correlation, r = −0.25, *p* = 0.013). This negative relationship was further illustrated when IgG2 levels were compared between asthmatic patients with ≤1.8 IU/L or >1.8 IU/L β-HEX. The patients with the lower β-HEX levels had significantly higher levels of serum IgG2 (Mann Whitney, *p* = 0.0016) 

## 4. Discussion

Asthma is a complicated condition mediated by gene and environment interactions that result in a wide range of outcomes in the population. Biomarkers have proven useful for asthma endotyping and support of personalized care [35]. To improve biomarker profiles, we tested plasma factors in our pediatric cohort of severe asthmatics in Memphis. Compared to both hospitalized nonasthmatics and healthy children, children with asthma had significantly elevated levels of periostin, a pleiotropic cytokine that promotes eosinophil recruitment and tissue remodeling in asthma [36]. Asthmatics also had significantly higher levels of β-HEX and IgE compared to hospitalized controls. IgG1, IgG2 and IgA levels were significantly lower in asthmatics compared to controls, and were often below age-specific reference ranges, consistent with some previous reports [37,38,39,40]. Vitamin A deficiencies/insufficiencies, as defined by low levels of the surrogate molecule RBP, were present in the majority of patients. For both asthmatic and hospitalized nonasthmatic children, RBP levels were significantly lower than previously reported levels among healthy children in the Memphis area [16].

Mannosyl-rich lysosomal hydrolases, such as β-HEX, are protease-resistant (and thus stable) in plasma until cleared by their receptors. A number of cell types such as mast cells, macrophages and T cells can secrete these enzymes [41,42]. We previously reported that β-HEX and its receptor (MRC2, calcium-dependent mannose receptor 2) have a putative role in airway smooth muscle remodeling [41,42,43]. Moreover, the MRC2 receptor blocker mannan, derived from *Saccharomyces cerevisiae*, effectively inhibits inflammation and airway smooth muscle remodeling in a humanized MRC2-overexpressing mouse model of allergic asthma [41,43]. Tomasiak et al. have reported elevated plasma β-HEX in adult asthmatics, particularly in patients with severe disease [19]. 

As basophils and mast cells can produce β-HEX, IL-4 and IL-5 after allergen stimulation, and secreted enzymes such as tryptase can induce the release of β-HEX from eosinophils in the blood [44], these molecules may engage in both the induction and exacerbation of asthma through positive feedback loops. β-HEX’s positive association with IL-5/IL-13 (known promoters of the Th2 response), negative association with IGFBP-1 and IgG2, elevated stability in plasma, and low cost of measurement advocate for the use of β-HEX as a routine biomarker for severe asthma in pediatrics.

Our cytokine/chemokine data were consistent with previous findings that periostin can be upregulated in severe asthmatics [45,46,47,48,49]. The importance of RAGE in allergic inflammation has been demonstrated in animal models [50,51,52] and single-nucleotide polymorphisms (SNPs) in RAGE were identified in patients with poor lung function [53,54], suggesting that higher levels of RAGE may either be a direct correlate or a proxy for asthma severity. Eotaxin, a chemokine for eosinophils and typically elevated in allergic individuals as cells are induced by IL-4 and IL-13, was not elevated among severe asthmatics in our study compared to hospitalized pediatric controls [55,56,57,58]. Previous studies have yielded variable results for eotaxin measurements in asthmatic patients on corticosteroid treatments [59,60,61], suggesting that this factor may not serve as a clear biomarker for severe asthma.

One highly significant finding in our study was that severe asthmatics had reduced IGFBP-1, a permissive condition for increased free IGF-1, both in comparison to hospitalized nonasthmatics and healthy children. This finding, together with previous reports of IGF-1 involvement in asthma pathogenesis [62,63] suggest that IGF-1 may provide an important target for asthma therapy. 

As stated above, our study identified low levels of RBP in the majority of our cohort, a finding that supports previous studies of vitamins and diets [64,65,66,67], including a meta-analysis that revealed a negative correlation between vitamin A and asthma frequency/severity [68,69,70,71,72]. Relationships between RBP and serum immunoglobulins or antibody responses have been demonstrated [73]. As mentioned previously, baseline RBP values correlated positively with the immune response towards an influenza virus vaccine in a recent pediatric study [16]. Similarly, RBP associations with influenza virus-specific neutralizing antibodies and serum immunoglobulin isotypes IgG4 and IgA have been demonstrated [73]. The noted positive correlation between RBP and IgG1 in this study further emphasizes the importance of vitamin A for the development and maintenance of a healthy immune system.

What is the cause-effect relationship between asthma, low RBP/retinol and low immunoglobulins? A simple explanation for low RBP may be that RBP is an acute phase protein that is reduced in the context of severe disease [21]. In addition, immunosuppressive treatments (corticosteroids, bronchodilators, omalizumab, immunotherapies) administered during exacerbations are expected to reduce responses by basophils [4,74], mast cells [75], and lymphocytes. Some asthmatics are steroid-resistant [76,77] while others require increasing doses of immunosuppressive drugs over time. Therefore, it is possible that chronic use of immunotherapies increases side-effects including a progressive decline in immune function, particularly in children [78,79,80]. 

An alternative, but nonmutually-exclusive hypothesis to explain cause-effect relationships is considered here. We note that low RBP levels are frequent among children and adults in Memphis [16,17,73]. When vitamin A levels are insufficient/deficient, total blood immunoglobulin levels and pathogen-specific antibody responses are reduced [16,29,31,32,33,34,73,81,82]. Low vitamin A levels can also lead to weaknesses in barrier functions provided by airway epithelial cells [83]. Here, we propose that low levels of vitamin A among Memphian children reduce immune and barrier functions to render individuals susceptible to airway diseases [84,85]. A combination of inert particles (e.g., pollen, smoke) and respiratory pathogens may then predispose children to asthma [86]. A corollary of this hypothesis is that an improvement in vitamin A intake among children could provide a simple prophylactic measure against asthma pathogenesis in Memphis.

This study had several limitations. First, we were unable to enroll healthy children at the same time as asthmatics in the hospital setting. In most cases, our study used only hospitalized, nonasthmatic children as controls and should be interpreted in that context. These hospitalized nonasthmatics had a variety of diseases/conditions that likely influenced immune markers. The bias toward African Americans and males in the test group, compared to controls, may have also lent to differences. Both test and control groups received a variety of drugs (including anti-IgE and other immunosuppressive drugs, insulin and antiepileptic drugs) that likely had a significant impact on the plasma biomarkers measured. Our study would have been strengthened if nutritional metrics had been collected on each patient in parallel with RBP analyses, and if studies of cells and mucosal tissues had been performed in parallel with our studies of plasma. Finally, we note that IL-4 was not measured in our study. This cytokine can be released by basophils and mast cells to trigger an early Th2 bias [44,87,88,89]. Nonetheless, our experiments identified unique biomarker patterns that characterized children during treatment for severe asthma. The continued assembly of biomarker profiles for severe asthma may ultimately support personalized treatments to prevent disease progression in asthmatic children.

## 5. Conclusions

This study illustrated unique features of patients with pediatric asthma in Memphis, TN. Asthmatics exhibited significantly higher levels of periostin, SP-D, RAGE and β-Hex compared to hospitalized, nonasthmatic controls. In contrast, IGFBP-1, eotaxin and granzyme A were significantly reduced in asthmatics compared to controls. Children with severe asthma also had lower levels of IgG1, IgG2, and IgA, and higher levels of IgE, compared to controls, and approximately half of the asthma patients exhibited IgG1 levels that were below age-specific norms. Vitamin A levels were insufficient or deficient in most asthmatic children and correlated positively with IgG1. Results encourage further evaluations of biomarkers in asthmatic children and an ongoing assessment of nutrition in vulnerable populations. If we consider that vitamin A deficiencies/immunodeficiencies may predispose children to infection, respiratory tissue damage and asthma, we may also consider that improvements in vitamin A intake may help prevent disease. Attention to biomarkers and nutrition may enhance diagnostics and the development of treatment options to improve disease outcomes in pediatric patients suffering from asthma. 

## Figures and Tables

**Table 1:** Patient Characteristics.

	Asthmatics	Nonasthmatics
	(Total *n* = 95)	(Total *n* = 47)
Age (years)		
3–6	25	13
7–12	42	13
13–18	28	21
Mean age (±SD)	9.86 (±4.09)	10.43 (±4.28)
Gender		
Male	70	22
Female	25	25
Race		
African American	82	30
White	9	15
Hispanic	2	0
Asian	1	1
Other	0	1
Undeclared	1	0
Therapy at Enrollment		
Chronic Oral Steroids	11	0
High Dose ICS (LABA)	84 (45)	0
Montelukast	59	0
Omalizumab	6	0
Allergen Immunotherapy	16	0
Anticholinergic	1	0
Other	0	ATB, Insulin, anti-epileptic
History of ICU Admission	62	0
#Asthma-Related Emergency Room Visits		
0–4	76	0
5–10	12	0
>10	7	0
Reported Smoke Exposure	22	Unknown

SD = Standard deviation, ICS = inhaled corticosteroids, LABA = Long-Acting β2-Agonist.

## Supplementary Materials

The following are available online at https://www.mdpi.com/2227-9059/8/10/393/s1, Supplementary Materials Figure S1. Immunoglobulin levels by age group. Immunoglobulin levels were grouped by age. Arrows define the lower limit (LL) of the age-specific reference ranges (Mayo Clinic) for each group. Reference ranges for IgE are not shown due to different age brackets (See Supplementary Materials Table S1). Supplementary Materials Figure S2. RBP levels by age group. RBP values were grouped by age to match the grouped immunoglobulin levels in Supplementary Materials Figure S1.

## Abbreviations

Retinol binding protein (RBP); β-hexosaminidase (β-HEX); limit of detection (LOD); receptor for advanced glycation end products (RAGE); interleukin (IL); insulin growth factor binding protein (IGFBP); interferon (IFN); enzyme-linked immunosorbent assay (ELISA); surfactant protein-D (SP-D); immunoglobulin (Ig); vitamin A deficiency (VAD); standard deviation (SD); inhaled corticosteroids (ICS); long-acting β2-agonist (LABA); Tennessee (TN); micrograms (mcg); milligram per kilogram (mg/kg); twice a day (bis in die, BID); single-nucleotide polymorphism (SNP); calcium-dependent mannose receptor 2 (MRC2), confidence interval (CI).
