# Supplementary material for: Questioning Cause and Effect: Children with Severe Asthma Exhibit High Levels of Inflammatory Biomarkers Including Beta-Hexosaminidase, but Low Levels of Vitamin A and Immunoglobulins
Source: Biomedicines. 2020 Oct 6;8(10):393. doi: 10.3390/biomedicines8100393 (PMC7600116; doi:10.3390/biomedicines8100393)
Supplement: Supplementary file 1 [file biomedicines-08-00393-s001.pdf]

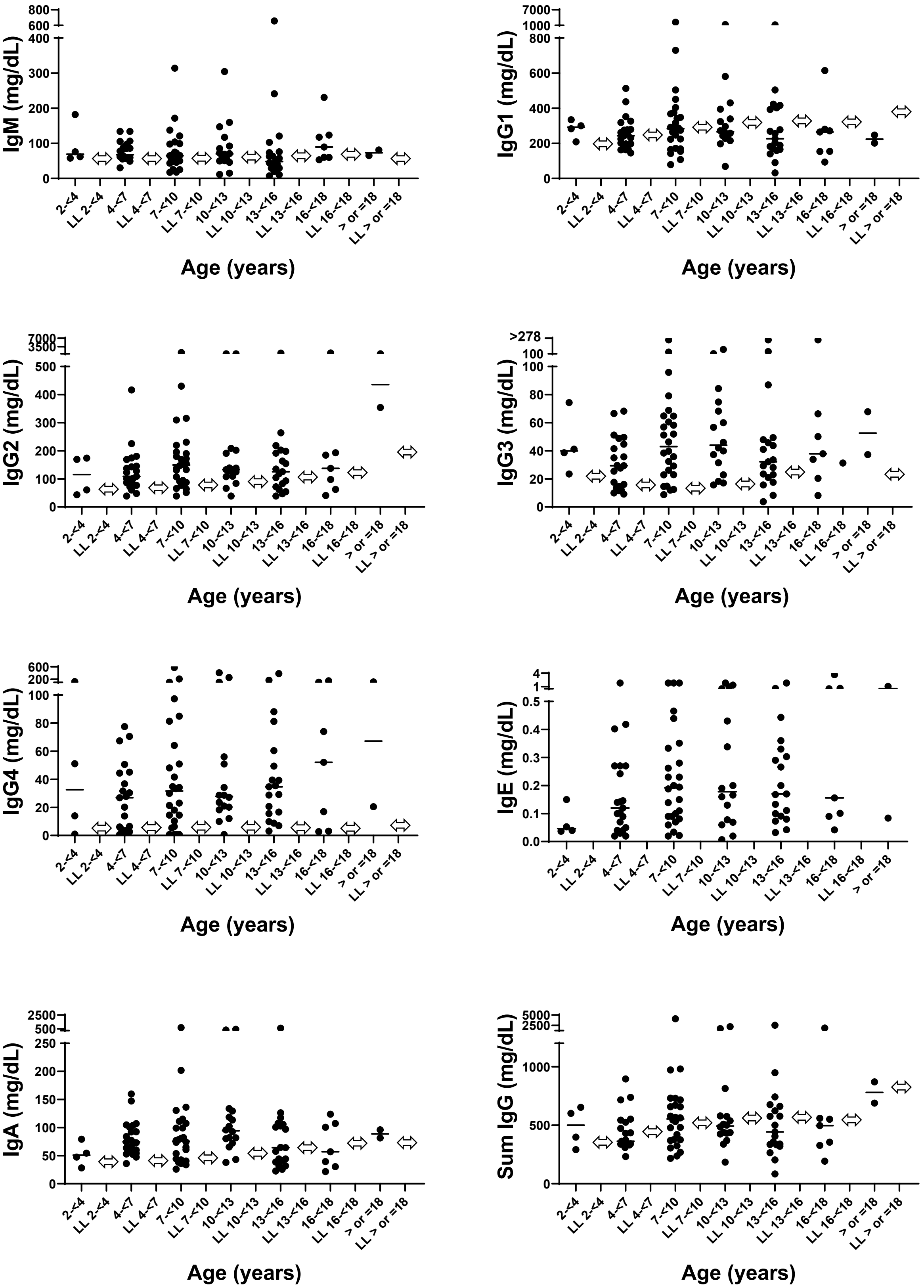
IgM (mg/dL)
IgG1 (mg/dL)
IgG2 (mg/dL)
IgG3 (mg/dL)
IgG4 (mg/dL)
IgE (mg/dL)
IgA (mg/dL)
Sum IgG (mg/dL)
Age (years)
2-<4
LL 2-<4
4-<7
LL 4-<7
7-<10
LL 7-<10
10-<13
LL 10-<13
13-<16
LL 13-<16
16-<18
LL 16-<18
> or =18
LL > or =18
>278

Supplementary Materials Figure S1 . Immunoglobulin levels by age group. Immunoglobulin levels were grouped by age. Arrows define the lower limit (LL) of the age-specific reference ranges (Mayo Clinic) for each group. Arrows define the lower limit (LL) of the age-specific reference ranges (Mayo Clinic) for each group. Reference ranges for IgE are not shown due to different age brackets (See Supplementary Materials Table S1).

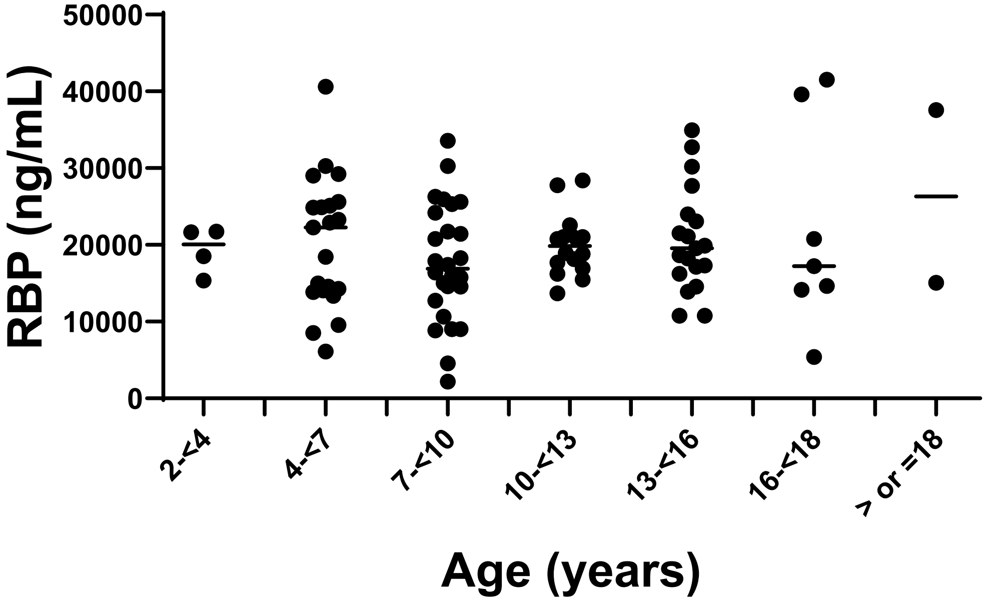

Supplementary Materials Figure S2. RBP levels by age group. RBP values were grouped by age to match the grouped immunoglobulin levels in Supplementary Materials Figure S1.

Supplementary Table 1: Immunoglobulin 95% Confidence Intervals and Reference Intervals by Age

	Total IgG	IgA	IgM	IgG1	IgG2	IgG3	
Age	95% CI	95% CI	95% CI	95% CI	95% CI	95% CI	9
0-<5 months	100-334 mg/dL	7-37 mg/dL	26-122 mg/dL	56-215 mg/dL	< or =82 mg/dL	7.6-82.3 mg/dL	< c r
5-<9 months	164-588 mg/dL	16-50 mg/dL	32-132 mg/dL	102-369 mg/dL	< or =89 mg/dL	11.9-74.0 mg/dL	< c r
9-<15 months	246-904 mg/dL	27-66 mg/dL	40-143 mg/dL	160-562 mg/dL	24-98 mg/dL	17.3-63.7 mg/dL	< c r
15-<24 months	313-1,170 mg/dL	36-79 mg/dL	46-152 mg/dL	209-724 mg/dL	35-105 mg/dL	21.9-55.0 mg/dL	< c r
2-<4 years	295-1,156 mg/dL	27-246 mg/dL	37-184 mg/dL	158-721 mg/dL	39-176 mg/dL	17.0-84.7 mg/dL	0.4-4
4-<7 years	386-1,470 mg/dL	29-256 mg/dL	37-224 mg/dL	209-902 mg/dL	44-316 mg/dL	10.8-94.9 mg/dL	0.8-8

7-<10 years:	462-1,682 mg/dL	34-274 mg/dL	38-251 mg/dL	253-1,019 mg/dL	54-435 mg/dL	8.5-102.6 mg/dL	1.[illegible] r[illegible]
10-<13 years	503-1,719 mg/dL	42-295 mg/dL	41-255 mg/dL	280-1,030 mg/dL	66-502 mg/dL	11.5-105.3 mg/dL	1.[illegible] r[illegible]
13-<16 years	509-1,580 mg/dL	52-319 mg/dL	45-244 mg/dL	289-934 mg/dL	82-516 mg/dL	20.0-103.2 mg/dL	0.[illegible] r[illegible]
16-<18 years	487-1,327 mg/dL	60-337 mg/dL	49-201 mg/dL	283-772 mg/dL	98-486 mg/dL	31.3-97.6 mg/dL	0.[illegible] r[illegible]
> or =18 years	767-1,590 mg/dL	61-356 mg/dL	37-286 mg/dL	341-894 mg/dL	171-632 mg/dL	18.4-106.0 mg/dL	2.[illegible] r[illegible]

	**IgE**
**Age**	**Reference interval**
0-5 months	< or =13
6-11 months	< or =34
1 and 2 years	< or =97
3 years	< or =199
4-6 years	< or =307
7 and 8 years	< or =403
9-12 years	< or =696
13-<16 years	< or =629
16-<18 years	< or =537
> or =18 years	< or =214

Values derived from Mayo Clinic (mayocliniclabs.com, accessed October 5, 2020).
